# Biochar-Based Photothermal Hydrogel for Efficient Solar Water Purification

**DOI:** 10.3390/molecules28031157

**Published:** 2023-01-24

**Authors:** Liang Wang, Jilei Wei, Kun Fang, Chen Zhou, Shengyang Yang

**Affiliations:** 1 School of Chemistry and Materials Engineering, Nanjing Polytechnic Institute, 188 Xinle Road, Nanjing 210048, China; 2 Department of Chemistry and Chemical Engineering, Yangzhou University, 180 Siwangting Road, Yangzhou 225002, China; 3 Department of Physical Sciences, University of Central Missouri, Warrensburg, MI 64093, USA

**Keywords:** solar interface evaporation, seawater desalination, wastewater purification, AgNP@CC hybrid, ACPH membrane

## Abstract

The development of technology for solar interface evaporation has a significant meaning for the sustainable use of water resources in remote regions. However, establishing a solar evaporator with a high evaporation rate and favorable water treatment capabilities remains challenging. In this work, we reported a silver nanoparticle (AgNP)@carbonized cattail (CC)/polyvinyl alcohol (PVA) composite hydrogel (ACPH) membrane. Because of the successfully loaded AgNPs, which have a photothermal synergy with the CC, the ACPH-10 membrane obtained an excellent photothermal conversion performance. Additionally, the hydrophilicity of the ACPH-10 membrane ensures a sustainable water supply which is necessary for the improvement of the evaporation rate. Therefore, the ACPH-10 membrane achieves an evaporation rate of 1.66 kg m^−2^ h^−1^ and an efficiency of 88.0%, attributed to the remarkable photothermal conversion and water transmission. More importantly, the membrane exhibits superior purification ability in a variety of sewage. Pollutant removal rates in heavy metal and organic dye sewage have exceeded 99.8%. As a result, the ACPH membrane holds great promise for wastewater recovery and seawater desalination, which can aid in resolving the water crisis issue.

## 1. Introduction

A clean water supply is vitally important to human survival and development. Unfortunately, a shortage of freshwater has significantly threatened human health [1,2] due to the explosion of population and the progress of industrialization. Traditional water treatment technologies, such as membrane distillation [3,4], reverse osmosis [5,6], electrodialysis [7], and adsorption [8], have been used for freshwater production, but the significant manufacturing costs and energy requirements limit their application in poor districts. To this end, the solar interface evaporator has attracted popular interest in recent years in light of its low cost and utilizing renewable energy. Until now, the solar interface evaporator has demonstrated fantastic application prospects in the fields of seawater desalination and sewage purification [9,10,11]. Generally, a typical high-performance solar interface evaporator should have some unique features, such as outstanding light absorption performance, continuous and reliable water supply capacity, good thermal management ability, and porous steam channel structure [12,13,14,15]. In this respect, metal materials [16,17,18,19], semiconductors [20,21,22,23], carbon-based materials [24,25,26,27,28,29,30,31], and organic polymers [32,33] are the four broad categories of solar thermal conversion materials because of their high light absorption performance. Among these, biochar with high absorbance, high specific surface area, and stable chemical properties is regarded as an inexpensive and easily accessible carbon-based material. For example, a variety of biological materials, including wood [34,35], mushrooms [36], loofah [37], bamboo [38], and enteromorpha prolifera [39], have been employed to fabricate biochar, which serves as a photothermal material in the solar interface evaporator. However, the poor mechanical properties of biochar hinder its use on a large scale [40,41]. Different from biochar, gel material, which has a hydrophilic three-dimensional polymer network and favorable mechanical performance [42,43,44,45], is frequently used as the basic material for constructing solar interface evaporators due to its favorable water transportation, high salt tolerance, and resistance to pollution [46,47,48,49]. Regrettably, the poor light absorption property is inimical to the gel material used alone for solar water evaporation. Consequently, it is desired to find a combination of a three-dimensional gel network with other functional photothermal conversion materials to construct a solar interface evaporator.

As noble metal nanoparticles, Ag nanoparticles (AgNPs) can strongly absorb visible light due to the local surface plasmon resonance (LSPR) effect [15,50,51,52]. Moreover, AgNPs have excellent bactericidal properties and are therefore used in water purification. However, the absorption spectrum of AgNPs covers only part of the solar spectrum. To further extend the light absorption span, AgNPs are usually deposited onto other black materials, such as carbon-based materials. Cattail is a species of aquatic plant that is widely distributed in eastern Asia and Oceania. Due to its fluff microfiber structure, carbonized cattail has been found to possess favorable adsorption ability on organic contaminants in solution [40]. In addition, the favorable light absorption of carbonized cattail makes it a promising material for solar water purification. For this reason, the combination of AgNPs-deposited carbonized cattail and hydrogel can be expected as a feasible scheme for fabricating an efficient solar interface evaporator.

In this work, an AgNP@carbonized cattail/polyvinyl alcohol composite hydrogel (ACPH) membrane is developed as a solar interface evaporator for seawater desalination and wastewater treatment. First, AgNPs were photodeposited on carbonized cattail (CC) to prepare the AgNP@CC hybrid, which was then introduced into polyvinyl alcohol (PVA) hydrogel using a straightforward sol-gel approach and freeze-drying process. In the ACPH evaporator, AgNP@CC serves as a photothermal material for solar light absorption and thermal conversion; while the fibrous nanostructured CC and the hydrophilic PVA maintain a sustainable water supply. Moreover, the relatively low thermal conductivity of PVA limits the heat converted from light on the air–liquid interface, reducing the heat loss to the bulk water. Based on this design, the ACPH membrane demonstrates a water evaporation rate of up to 1.66 kg m^−2^ h^−1^ with a photothermal conversion efficiency of 88.0% under 1 sun. Moreover, the ACPH membrane was employed for solar-driven seawater desalination and wastewater purification. The results reveal that the salts, heavy metal ions, and organic dyes are almost completely removed after evaporation with the ACPH membrane. Therefore, it can be believed that the ACPH membrane shows a great potential application for solar water purification.

## 2. Experimental Section

### 2.1. Materials

Cattail was picked from Liaojiagou City Central Park of Yangzhou (Jiangsu, China). Silver nitrate (AgNO_3_, 99.8%) and magnesium sulfate anhydrous (MgSO_4_, AR) were purchased from Aladdin Chemical Reagent Co., Ltd. (Shanghai, China). Methyl orange (C_14_H_14_N_3_NaO_3_S, 96%), methylene blue (C_16_H_18_N_3_SCl, AR), Rhodamine B (C_28_H_31_ClN_2_O_3_, 99.0%), lead(II) acetate trihydrate (Pb(CH_3_COO)_2_·3H_2_O, 99.99%), chromium acetate (Cr(CH_3_COO)_3_, 99.9%), and cadmium acetate dihydrate (Cd(CH_3_COO)_2_·2H_2_O, 99.99%) were purchased from Shanghai Macklin Biochemical Co., Ltd. (Shanghai, China). Polyvinyl alcohol (PVA–1788), hydrochloric acid (HCl, 37.0%), ethanol (C_2_H_6_O, 99.7%), glutaraldehyde (C_5_H_8_O_2_, 50.0%), sodium chloride (NaCl, AR), potassium chloride (KCl, AR), and calcium chloride anhydrous (CaCl_2_, AR) were purchased from Sinopharm Chemical Reagent Co., Ltd. (Shanghai, China). Ultrapure water with an electrical resistivity of 18.2 MΩ·cm was utilized as the experiment water.

### 2.2. Preparation of AgNPs@Carbonized Cattail (AgNP@CC) Hybrid

The cleaned and dried cattail was carbonized in a tubular furnace (OTF-1200X-S, Hefei Kejing Material Technology Co., Ltd., Hefei, Anhui, China) at 500 °C under a *N_2_* atmosphere. After that, the obtained carbonized cattail (CC) was ground into a fine powder. CC powder (2 g) was dispersed in an ethanol aqueous solution (50 mL, 25 wt%) by ultrasonication. Subsequently, 0.1 g of silver nitrate was added to the solution under sonication. The mixture was then placed into a Parker reactor for photodeposition reaction (2 h, magnetic stirring) with a Xe lamp (CEL-HXF300, Zhongjiao Jinyuan Technology Co., Ltd., Beijing, China) under vacuum. The AgNP@CC prepared by photodeposition was repeatedly washed to eliminate impurities with pure water (3 times) by centrifuge (H1650R, Hunan Xiangyi Laboratory Instrument Development Co., Ltd., Changsha, Hunan, China) and finally dried under vacuum for further use.

### 2.3. Preparation of AgNP@CC/PVA Hydrogel (ACPH) Membrane

Different weights of AgNP@CC (0 g, 0.005 g, 0.0125 g, 0.025 g, and 0.05 g) were separately added to PVA solution (5 g, 5 wt%) and stirred for 30 min. Then, 80 μL of glutaraldehyde (50 wt%) and 250 μL of HCl solution (37 wt%) were added to each PVA mixture and thoroughly stirred to form AgNP@CC/PVA hydrogels. Subsequently, the hydrogels were soaked in ultrapure water for 12 h, followed by lyophilization to fabricate ACPH membranes with various amounts of AgNP@CC. These AgNP@CC/PVA hydrogel membranes were named ACPH-0, ACPH-2, ACPH-5, ACPH-10, and ACPH-20, respectively, where the number indicates the weight ratio of AgNP@CC to PVA.

### 2.4. Solar-Driven Water Evaporation with ACPH Membrane

The solar-driven water evaporation experiment was conducted in a quartz cup with a diameter of 4 cm under simulated sunlight. For the simulation of sunlight, a Xe lamp (CEL-HXF300, Zhongjiao Jinyuan Technology Co., Ltd., Beijing, China) with an AM 1.5 G filter was employed and the light intensity was fixed at 1 kW m^−2^. Before the experiment, the ACPH membrane was embedded in EPE foam with a circular ring shape (inner diameter: 2.4 cm, outer diameter: 4 cm). The evaporation experiment was carried out in a closed room in which the temperature and the relative humidity were controlled at about 28 °C and 30%, respectively. The evaporation rate was calculated based on the weight loss of the water, which was measured by the electronic balance with an accuracy of 0.1 mg (LE204E, Mettler-toledo Apparatus Co., Ltd., Shanghai, China).

### 2.5. Solar-Driven Seawater Desalination and Wastewater Purification

The experimental device for seawater desalination and wastewater purification was similar to that described in the water evaporation test except a closed clear quartz collector was involved. The ion concentrations of Na^+^, Mg^2+^, K^+^, and Ca^2+^ in simulated seawater are 10,780 mg L^−1^, 1298 mg L^−1^, 400 mg L^−1^, and 410 mg L^−1^, respectively. To investigate the desalination performance of the ACPH membrane, the distilled water was collected and detected by the inductively coupled plasma mass spectrometer. Two different concentrations (10 and 100 mg L^−1^) of heavy metal (Cr^2+^, Cd^2+^, and Pb^2+^) wastewater were prepared for the purification experiment. Similar to the seawater desalination experiment, the solar-driven heavy metal wastewater treatment begins with the same steps and ends with the detection of remaining pollutants in the evaporated water. Methyl orange, methylene blue, and Rhodamine B with a concentration of 10 ppm were chosen as the organic pollutants to evaluate the purification performance of the ACPH membrane. The change in concentration was detected by high-performance liquid chromatography.

### 2.6. Characterization

Scanning electron microscopy (SEM) images of CC, AgNP@CC, and ACPH membrane were obtained with a field-emission scanning electron microscope system (GeminiSEM 300, Carl Zeiss, Oberkochen, Germany). Transmission electron microscopy (TEM) images of AgNP@CC were obtained with a field-emission transmission electron microscope at 300 kV (Tecnai G2 F30 S-TWIN, Philips-FEI, Netherlands). X-ray diffraction (XRD) measurements of ACPH membrane and combined material AgNP@CC were completed on an X-ray diffractometer (D8 Advance, BRUKER-AXS, Karlsruhe, Germany) with Cu Kα radiation. The Fourier transform infrared spectra (FT-IR) of raw cattail and CC were obtained using a Fourier transform infrared spectrometer (Tensor II, Bruker Optics GmbH, Ettlingen, Germany) from 400 to 4000 cm^−1^. UV-Vis spectra and solid UV-Vis diffuse reflectance spectra were obtained using a UV-Vis spectrophotometer (i5, Hanon, Jinan, China) and a UV-Vis-NIR spectrophotometer (Cary-5000, Varian, Inc., Palo Alto, CA, USA), respectively. The thermal conductivity and thermal diffusion coefficient were obtained by a transient plane heat source method thermal conductivity meter (HCDR-S, Huicheng, Nanjing, China). Digital infrared thermal imaging was observed by a thermal infrared imager (TESTO 869, TESTO SE & Co. KGaA, thermal Imager, Germany). The concentration of ions was detected by an inductively coupled plasma mass spectrometer (XSeries II, Thermo Fisher, MA, USA). The contents of methyl orange, methylene blue, and Rhodamine B were determined by high-performance liquid chromatography (HPLC1260, Agilent, Agilent Technologies, CA, USA) and ultraviolet-visible spectrophotometer (i5, Hanon, Jinan, China).

### 2.7. Calculation of Water Evaporation Efficiency

The solar-to-vapor conversion efficiency (η) is calculated by the formula [53,54]:

(1) $$\eta =\frac{q_{m}\left(C_{p}\times \Delta T+h_{lv}\right)}{P_{i}}$$

where *q_m_* is the evaporation rate under solar illumination (kg m^−2^ h^−1^), *C_p_* is the specific heat capacity of water and a constant of 4.18 kJ kg^−1^ K^−1^, Δ*T* indicates the increased temperature of the absorber surface (K), *P_i_* is the power density of solar illumination on the ACPH membrane (1 kW m^−2^), and *h_lv_* is the actual evaporation enthalpy of water (kJ kg^−1^), which can be estimated by the following equation (Equation (2)).

(2) $$h_{lv}=\frac{h_{0}}{m_{r}}$$

where *h_0_* represents the latent enthalpy of water at a certain temperature (kJ kg^−1^), and *m_r_* represents the ratio of the natural evaporation rate of the ACPH membrane to the evaporation rate of the blank pure water sample under the dark state.

## 3. Results and Discussion

**Figure 1:**
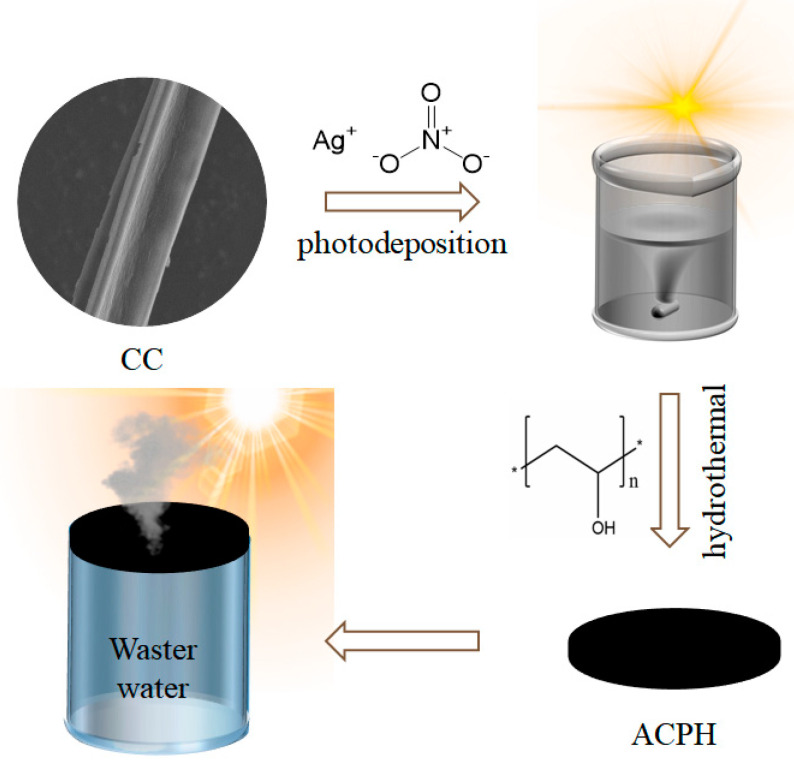
Schematic illustration of the synthesis procedure of the ACPH membrane and its application for solar water purification.

The preparation process of the ACPH membrane is shown in Figure 1. Firstly, the AgNPs were doped to the CC by a photodeposition process to synthesize the AgNP@CC. The AgNP@CC was then extensively mixed with PVA solution and freeze-dried to create the ACPH membrane for desalination and wastewater purification. The detailed experimental process for the production of the ACPH membrane can be found in the Section 2. Raw cattail and CC were both subjected to an FT-IR analysis to determine their functional groups (Figure S1). The stretching vibrations of the -OH, -CH_2_, and six-membered cyclic ether bonds, respectively, are represented by the absorption peaks near 3385, 2920, and 1044 cm^−1^ in the FT-IR spectra of raw cattail. Aldehyde, carboxyl, ketone, carbonyl, and ester group stretching vibrations are primarily responsible for the absorption characteristic peaks at 1735, 1609, and 1516 cm^−1^. The majority of the -OH and C-O groups are still present in CC, which ensures the outstanding hydrophilicity of the obtained CC. Furthermore, the numerous functional groups in CC boost the water absorption capability of the ACPH membrane, which will be beneficial for water purification.

**Figure 2:**
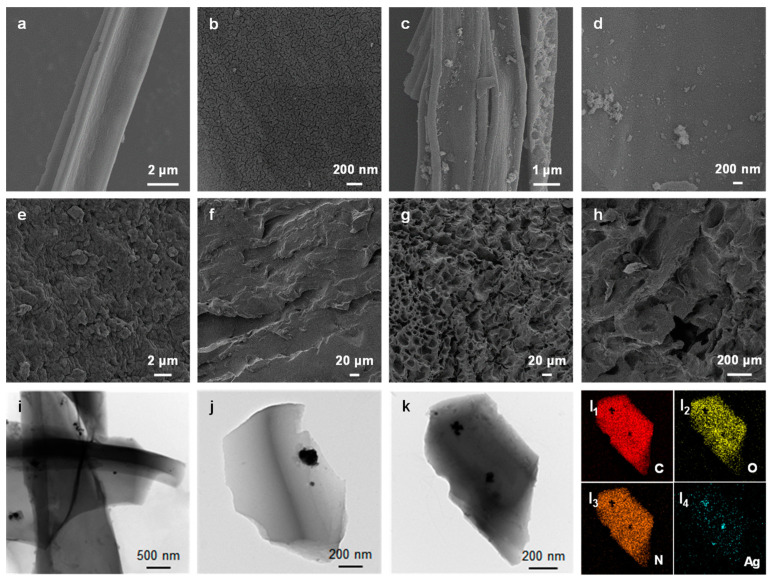
SEM image of (**a**,**b**) CC, (**c**,**d**) AgNP@CC, (**e**) top-view of the ACPH-0 membrane, (**f**) cross-section of the ACPH-0 membrane, (**g**) top-view of the ACPH-10 membrane, and (**h**) cross-section of the ACPH-10 membrane. (**i**–**k**) TEM images of AgNP@CC. (**l**) EDS element distribution maps of C, O, N, and Ag in AgNP@CC.

SEM was used to examine the morphology and microstructure of the CC, AgNP@CC, ACPH-0 membrane, and ACPH-10 membrane. The plant multi-fiber of the cattail was retained after the calcination process and a lot of flakes were observed on the surface of the fiber (Figure 2a,b). Interestingly, these flake structures increased the contact area and provided abundant attachment sites for the generation of the AgNPs. As shown in Figure 2c,d, the AgNPs were uniformly distributed on the surface of the CC. The PVA hydrogel membrane without the combined material AgNP@CC (ACPH-0) contains fewer pores with smaller sizes on the surface, and some of the pores were closed from the cross-section, resulting in a tighter overall structure (Figure 2e,f). In contrast, the internal structure of the membrane became loose and porous after mixing with the AgNP@CC (Figure 2g,h). Therefore, the ACPH-10 membrane can maintain a sustainable water supply and effective steam transmission due to its porous structure. The TEM images of the AgNP@CC indicated that the AgNPs were generated on the surface of the CC successfully as expected (Figure 2i–k). Energy-dispersive spectroscopy (EDS) study of the element distribution reveals that the C, O, and N elements are uniformly dispersed, and AgNPs are evenly scattered on the surface of CC, which further supports the effective synthesis of combined material AgNP@CC (Figure 2l). In addition, the energy dispersive spectrum analysis of the AgNP@CC revealed that the content of Ag is low, which reduced the preparation cost of the ACPH-10 membrane (Figure S2).

**Figure 3:**
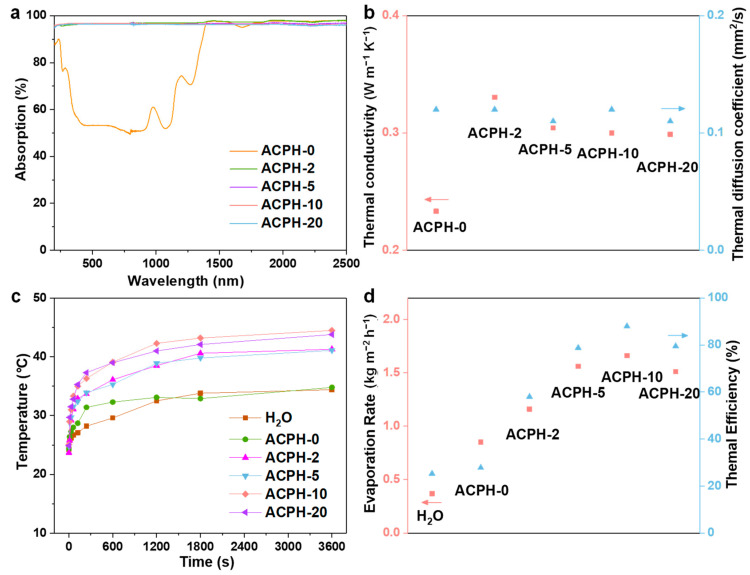
The (**a**) UV-Vis-NIR absorption spectra and (**b**) thermal conductivity and diffusion coefficient of the ACPH-0, ACPH-2, ACPH-5, ACPH-10, and ACPH-20 membranes, respectively. (**c**) The surface temperature curves of the H_2_O, ACPH-0, ACPH-2, ACPH-5, ACPH-10, and ACPH-20 membrane under 1 sun irradiation for 1 h. (**d**) Water evaporation rate and thermal efficiency of the H_2_O, ACPH-0, ACPH-2, ACPH-5, ACPH-10, and ACPH-20 membranes.

The UV-Vis-NIR absorption spectra can intuitively reflect the light absorption ability of the sample. Therefore, the absorption spectra in the range of 200–2500 nm of different membranes are described in Figure 3a. In the spectral region of 200–2500 nm, the average absorbance of the ACPH-0 membrane is about 78.76%, while this value for the ACPH-20 membrane can reach up to 95.8%. This demonstrates that the addition of AgNP@CC improves light absorption effectively. In addition, the thermal insulation property is an important factor that impacts the evaporation performance of the membrane. Therefore, the thermal conductivity and thermal diffusion coefficient of different membranes were measured (Figure 3b). The test results showed that the thermal diffusion coefficient of membranes is barely affected by the presence of AgNP@CC, which is probably related to the existence of the fundamental frame of PVA hydrogel. The thermal conductivity of the membranes decreases gradually with the increase of the AgNP@CC content. Since the thermal conductivity of the ACPH-10 and ACPH-20 membranes is essentially the same, it is assumed that when the amount of AgNP@CC in the membrane increases, the thermal conductivity will eventually reach a constant value. Because of the favorable thermal insulation of membranes, which promotes heat accumulation and minimizes heat loss, the surface temperature of ACPH-2, ACPH-5, ACPH-10, and ACPH-20 can reach up to 41.3 °C, 41.1 °C, 44.5 °C, and 43.8 °C, respectively (Figure 3c and Figures S3–S8). The highest temperature differential (19.4 °C) and the fastest rate of temperature rise in the evaporation process among the five membranes are found in the ACPH-10 membrane, indicating that it has the best photothermal performance. Therefore, the ACPH-10 membrane obtained the highest evaporation rate of 1.66 kg m^−2^ h^−1^ under 1 sun irradiation for 1 h, compared to the ACPH-0, ACPH-2, ACPH-5, and ACPH-20 membranes, which is 4.47 times that of pure water (0.37 kg m^−2^ h^−1^). Additionally, the corresponding solar-to-vapor conversion efficiency of the H_2_O, ACPH-0, ACPH-2, ACPH-5, ACPH-10, and ACPH-20 membranes was calculated at 25.3%, 32.4%, 58.1%, 78.8%, 88.0%, and 79.5%, respectively (Figure 3d and Table S1). Although the ACPH-20 membrane has the highest content of AgNP@CC, its evaporation performance is inferior to the ACPH-10 membrane, which is probably due to the excessive AgNP@CC in the ACPH membrane hindering water transportation.

**Figure 4:**
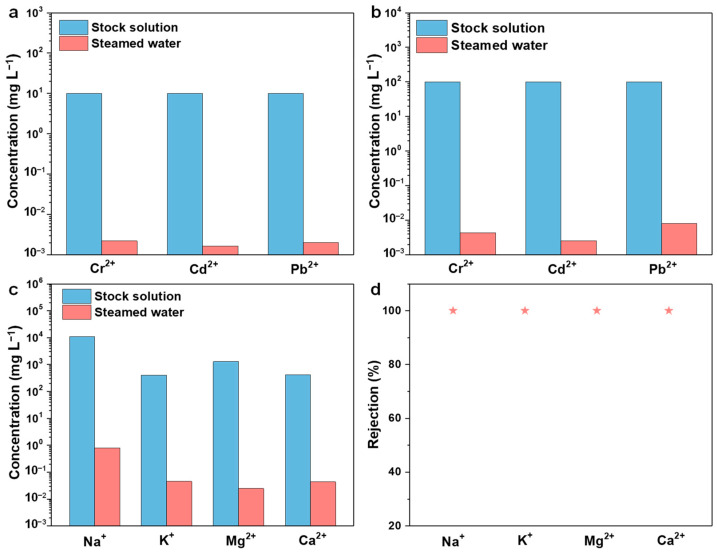
The concentrations of heavy metal ions with (**a**) 10 ppm and (**b**) 100 ppm before and after purification via the ACPH-10 membrane. (**c**) The concentrations of main metal ions in the simulated seawater before and after purification. (**d**) The rejection efficiency of each metal ion in the simulated seawater after purification.

The ecological environment and human health are significantly endangered by heavy metal water contamination. In order to determine the removal effectiveness of the ACPH-10 membrane from heavy metal wastewater with low concentration (10 mg L^−1^) and high concentration (100 mg L^−1^), water purification experiments were conducted. The wastewater purification experiment was carried out under 1 sun irradiation and the steamed water was collected to determine the residual concentration of the heavy metal ions (Cr^2+^, Cd^2+^, and Pb^2+^). For the low-concentration wastewater, the test revealed that the concentration of three heavy metal ions in the distilled water was only 2.21 μg L^−1^, 1.66 μg L^−1^, and 2.03 μg L^−1^, and the corresponding ion rejection efficiencies are 99.98%, 99.99%, and 99.98% (Figure 4a, Table S2). The ion rejection efficiencies of heavy metal ions for the high-concentration original solution were also over 99.99% (Figure 4b, Table S3). The level of heavy metal ions reduces by 4 and 5 orders of magnitude in steamed water from low-concentration (10 mg L^−1^) and high-concentration (100 mg L^−1^) wastewater, respectively, which is lower than the standard concentration advised by the World Health Organization (WHO) [54,55]. Therefore, it can be concluded that the ACPH-10 membrane has an excellent purification capability in heavy metal wastewater.

In recent years, seawater desalination has become one of the key strategies for addressing the water resource deficit. Therefore, in order to evaluate the desalination ability of the ACPH-10 membrane, a solar-powered seawater evaporation experiment was carried out. The simulated seawater which was prepared based on the concentration of major ions (Na^+^, Mg^2+^, K^+^, and Ca^2+^) in real seawater was employed for the desalination experiment. The concentrations of Na^+^, Mg^2+^, K^+^, and Ca^2+^ in the simulated seawater are 10,780 mg L^−1^, 1298 mg L^−1^, 400 mg L^−1^, and 410 mg L^−1^, respectively, while their concentrations in steamed water are lowered to 0.7952 mg L^−1^, 0.0246 mg L^−1^, 0.0451 mg L^−1^, and 0.0436 mg L^−1^, respectively (Figure 4c and Table S4). After purification with the ACPH-10 membrane, the removal of 99.99% for four metal ions was obtained, which meets the WHO drinking water quality standard. In addition, the concentration of the four ions in the steamed water is one or two orders of magnitude lower than that in most of the reported works [15,45,54,56,57,58,59], which makes the ACPH-10 membrane a satisfactory solar-powered evaporator (Table S5). Furthermore, there was no obvious salting-out phenomenon found during the test, which can be attributed to the natural dissolution [60,61].

**Figure 5:**
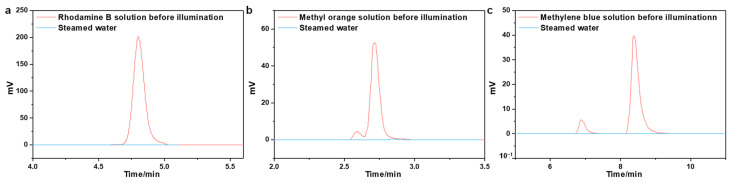
The High-performance liquid chromatographic spectra of (**a**) Rhodamine B, (**b**) methyl orange, and (**c**) methylene blue.

**Figure 6:**
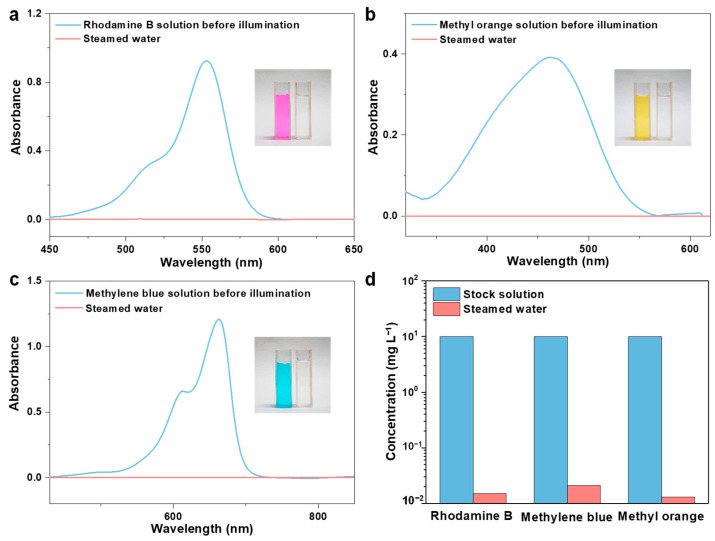
The UV-vis absorption spectroscopy of (**a**) Rhodamine B, (**b**) methyl orange, and (**c**) methylene blue solutions before and after purification. Inside: the photographs of contaminated water and steamed water after purification. (**d**) The change of concentration for three organic dye solutions before and after purification.

Organic pollutants are another primary source of water contamination that will accumulate in the environment and the human body, leading to a variety of adverse effects [62,63]. Therefore, we prepared an organic dye (Rhodamine B, methyl orange, and methylene blue) solution to investigate the decontamination performance of the ACPH-10 membrane. High-performance liquid chromatography (HPLC) was employed to measure the change in concentration of dyes before and after purification. The concentration of dyes decreased dramatically to 0.015 mg L^−1^, 0.021 mg L^−1^, and 0.013 mg L^−1^, respectively (Figure 5a–c). In comparison to the original solution, the color of the distilled solution has completely faded (Figure 6a–c), and the removal rate of three dyes has achieved about 99.8% (Figure 6d, Table S6).

## 4. Conclusions

In summary, we developed a silver nanoparticle (AgNP)@carbonized cattail (CC)/polyvinyl alcohol (PVA) composite hydrogel (ACPH) membrane for solar purification. The ACPH membrane has a three-dimensional polymer network structure, which is advantageous for water transport and evaporation. The photothermal performance of the ACPH membrane is favorable and steady as a result of the photothermal synergy between AgNPs and CC. In particular, the ACPH membrane has low thermal conductivity and strong light absorption, which can increase solar-to-vapor conversion efficiency while lowering heat loss from the system. Additionally, the hydrophilicity and adsorption properties of CC encourage the purification and evaporation of water. The ACPH-10-based evaporator’s evaporation rate and efficiency were the greatest of the six samples when tested under 1 sun, at 1.66 kg m^−2^ h^−1^ and 88.0%, respectively. Salts, organic dyes, and heavy metal ions were almost entirely eliminated when the ACPH membrane was used in solar-powered water treatment. This work offers a practical membrane material for solar-driven evaporation that is efficient, straightforward, and ecologically benign. The ACPH membrane is projected to be used for wastewater treatment and seawater desalination, which will help to address the freshwater scarcity in underdeveloped and distant locations.

## Data Availability

Data available on request to the corresponding author.

## Supplementary Materials

The following supporting information can be downloaded at: https://www.mdpi.com/article/10.3390/molecules28031157/s1, Table S1: The solar thermal conversion efficiency of H_2_O and ACPH membranes; Table S2: The ICP results of polluted water containing heavy metal ions before (c = 10 ppm) and after solar purification with the ACPH membrane; Table S3: The ICP results of polluted water containing heavy metal ions before (c = 100 ppm) and after solar purification with the ACPH membrane; Table S4: The ICP results of simulated seawater before and after solar purification with the PPPH-CF membrane; Table S5: Comparison of ion concentration in steamed water produced by evaporators of different materials; Table S6: The HPLC results of water samples containing dyes before and after solar purification with the ACPH membrane; Figure S1: FT-IR spectra of raw cattail and CC; Figure S2: The energy dispersive spectrum analysis of the AgNP@CC; Figure S3: Digital infrared thermal images of pure water; Figure S4: Digital infrared thermal images of pure water with the ACPH-0 membrane; Figure S5: Digital infrared thermal images of pure water with the ACPH-2 membrane; Figure S6: Digital infrared thermal images of pure water with the ACPH-5 membrane; Figure S7: Digital infrared thermal images of pure water with the ACPH-10 membrane; Figure S8: Digital infrared thermal images of pure water with the ACPH-20 membrane. References [15,50,54,56,57,58,59] are cited in the Supplementary Materials.

## Sample Availability

Samples of the compounds are not available from the authors.
