# Supplementary material for: Biochar-Based Photothermal Hydrogel for Efficient Solar Water Purification
Source: Molecules. 2023 Jan 24;28(3):1157. doi: 10.3390/molecules28031157 (PMC9919770; doi:10.3390/molecules28031157)
Supplement: Supplementary file 1 [file molecules-28-01157-s001.zip › molecules-2167120-supplementary.pdf]

Supplementary Material

Biochar-Based Photothermal Hydrogel for Efficient Solar Water Purification

Liang Wang [1], Jilei Wei [2], Kun Fang [2], Chen Zhou [2,3,*] and Shengyang Yang [2,*]

[1] School of Chemistry and Materials Engineering, Nanjing Polytechnic Institute, 188 Xinle Road, Nanjing 210048, China
[2] Department of Chemistry and Chemical Engineering, Yangzhou University, 180 Siwangting Road, Yangzhou 225002, China
[3] Department of Physical Sciences, University of Central Missouri, Warrensburg, MI 64093, USA
* Correspondence: zhou@ucmo.edu (C.Z.); syyang@yzu.edu.cn (S.Y.)

Table S1. The solar thermal conversion efficiency of H_2O and ACPH membranes.

Parameters	H_2O	ACPH-0	ACPH-2	ACPH-5	ACPH-10	ACPH-20
m_r	1	1.82	1.39	1.37	1.31	1.32
h_0 (kJ kg^{-1})	2419	2418	2402	2398	2395	2397
h_{lv} (kJ kg^{-1})	2419	1328	1728	1750	1828	1816
Evaporation rate (kg m^{-2} h^{-1})	0.37	0.85	1.16	1.56	1.66	1.51
Increased Temperature (K)	10.1	10.5	17.6	16.3	19.4	18.9
Efficiency (%)	25.3	32.4	58.1	78.8	88.0	79.5

Table S2. The ICP results of polluted water containing heavy metal ions before (c=10 ppm) and after solar purification with ACPH membrane.

Ions	Stock Solution (mg L^{-1})	Steamed Water (μg L^{-1})	Rejection Efficiency (%)
Cr^{2+}	10	2.21	99.98
Cd^{2+}	10	1.66	99.99
Pb^{2+}	10	2.03	99.98

Table S3. The ICP results of polluted water containing heavy metal ions before (c=100 ppm) and after solar purification with ACPH membrane.

Ions	Stock Solution (mg L^{-1})	Steamed Water (μg L^{-1})	Rejection Eficiency (%)
Cr^{2+}	100	4.32	99.99
Cd^{2+}	100	2.56	99.99
Pb^{2+}	100	8.20	99.99

Table S4. The ICP results of simulated seawater before and after solar purification with PPPH-CF membrane.

Ions	Stock Solution (mg L^{-1})	Steamed Water (mg L^{-1})	Rejection Efficiency (%)
Na^{+}	10780	0.7952	99.99
Mg^{2+}	1298	0.0246	99.99
K^{+}	400	0.0451	99.99
Ca^{2+}	410	0.0436	99.99

Table S5. Comparison of ion concentration in steamed water produced by evaporators of different materials

Evaporator	Na^{+} (mg L^{-1})	Mg^{2+} (mg L^{-1})	K^{+} (mg L^{-1})	Ca^{2+} (mg L^{-1})	References
Ag/PPy-PMBA-BrILs	~2	~0.5	~0.8	~1	[1]
HPH-based SVG	~6	0.95	~0.9	~0.9	[2]
rGOHM	~1	~0.3	~0.15	~0.4	[3]
SCG	~11	~2.3	~1.5	~8	[4]
CBS-Ti_3C_2	~5	~0.2	~0.8	~0.5	[5]
HHEs	~1.2	~0.2	~0.5	~0.4	[6]
SSC5	13.5	1.62	4.47	1.88	[7]
ACPH-10	0.7952	0.0246	0.0451	0.0436	**Our work**

Table S6. The HPLC results of water samples containing dyes before and after solar purification with ACPH membrane.

Dyes	Stock Solution (mg L^{-1})	Steamed Water (mg L^{-1})	Rejection Efficiency (%)
Rhodamine B	10	0.015	99.85
Methyl orange	10	0.021	99.79
Methylene blue	10	0.013	99.87

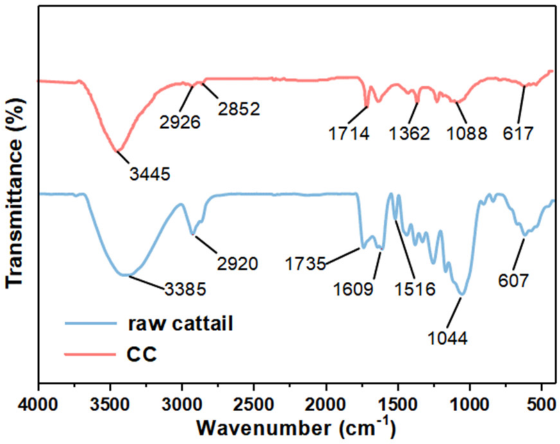

Figure S1. FT-IR spectra of raw cattail and CC.

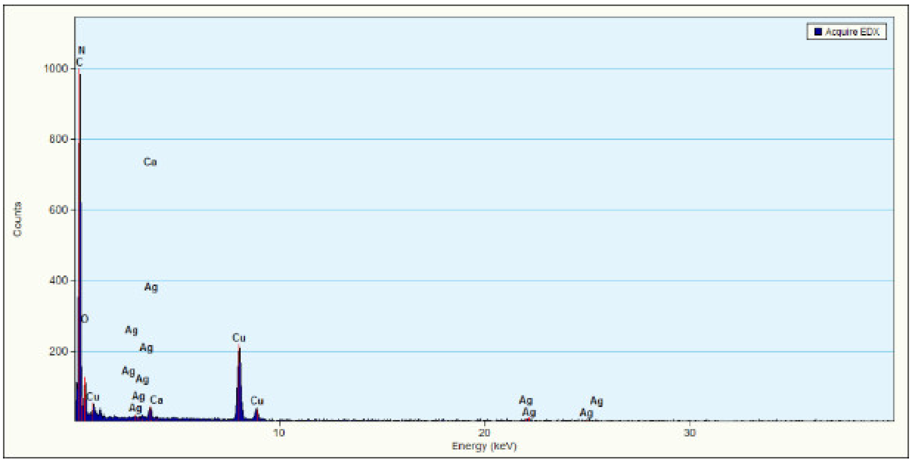

Figure S2. The energy dispersive spectrum analysis of the AgNPs@CC.

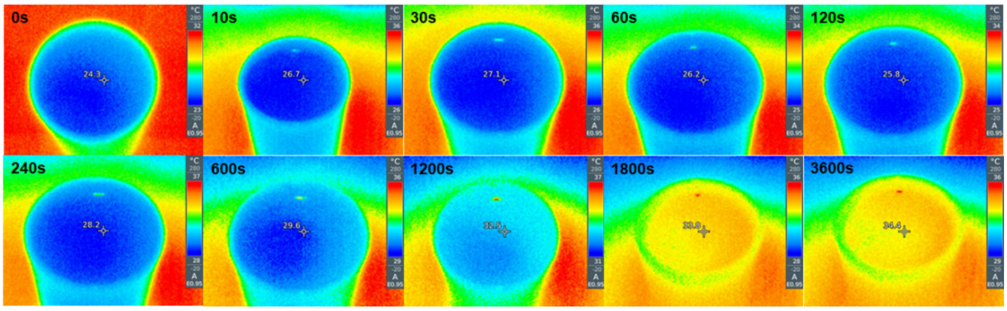

Figure S3. Digital infrared thermal images of pure water.

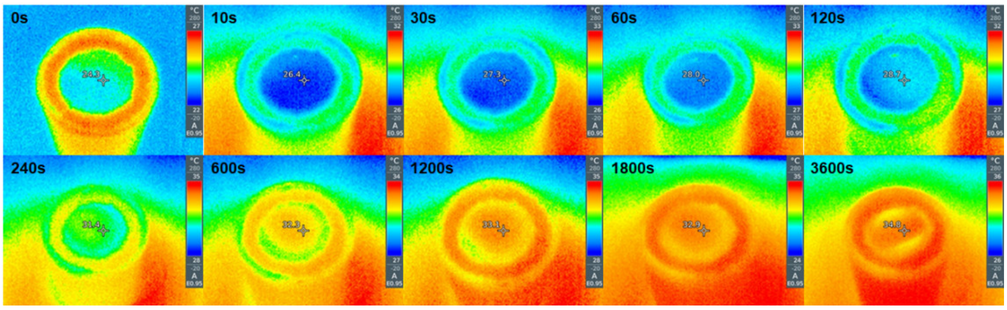

Figure S4. Digital infrared thermal images of pure water with ACPH-0 membrane.

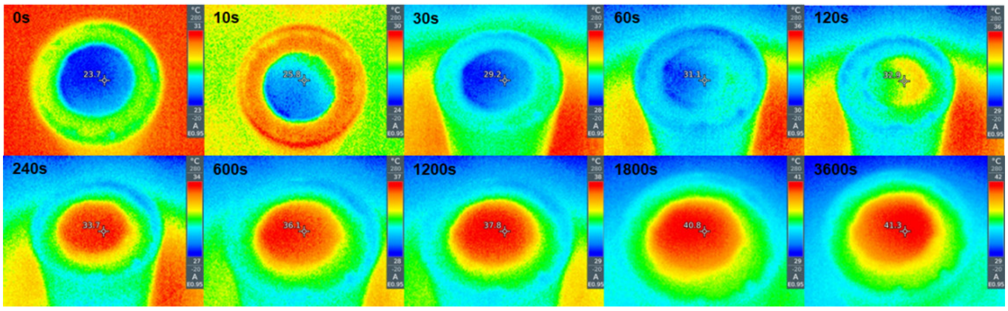

Figure S5. Digital infrared thermal images of pure water with ACPH-2 membrane.

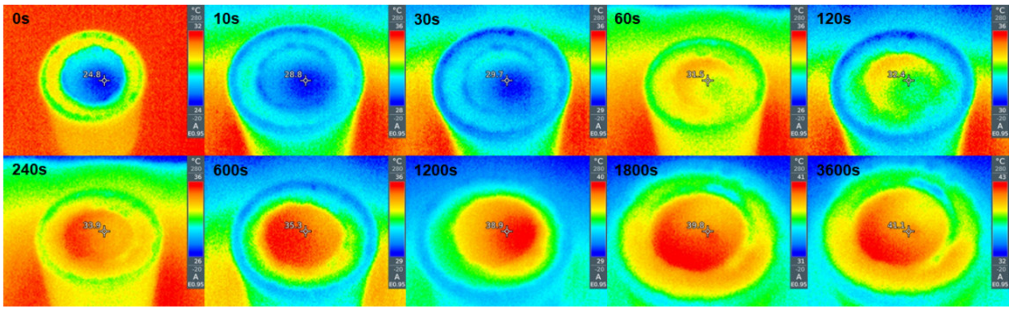

Figure S6. Digital infrared thermal images of pure water with ACPH-5 membrane.

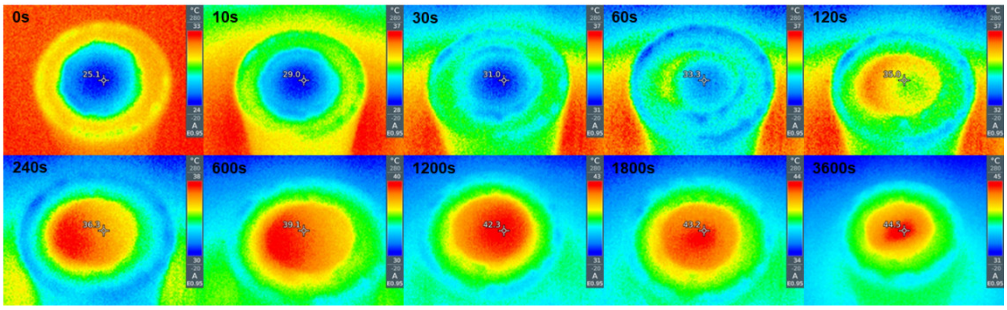

Figure S7. Digital infrared thermal images of pure water with ACPH-10 membrane.

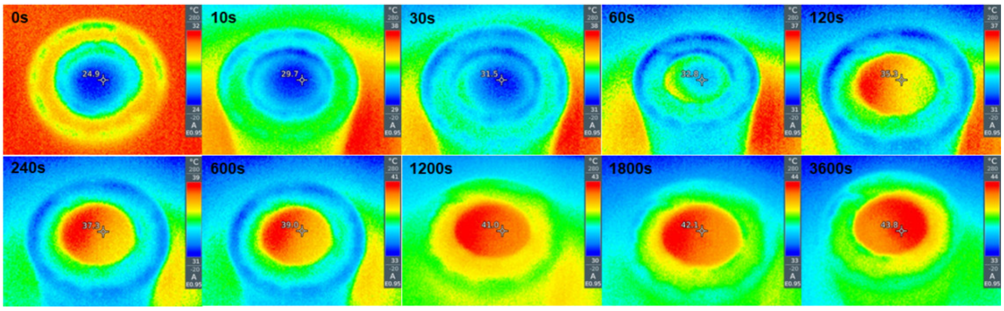

Figure S8. Digital infrared thermal images of pure water with ACPH-20 membrane.

References

1. Zhao, L.; Du, C.; Zhou, C.; Sun, S.; Jia, Y.; Yuan, J.; Song, G.; Zhou, X.; Zhao, Q.; Yang, S. Structurally ordered AgNPs@C_3N_4/GO membranes toward solar-driven freshwater generation. *ACS Sustain. Chem. Eng.* **2020**, *8*, 4362–4370.
2. Xiao, C.; Liang, W.; Hasi, Q.; Chen, L.; He, J.; Liu, F.; Wang, C.; Sun, H.; Zhu, Z.; Li, A. Ag/polypyrrole co-modified poly(ionic liquid)s hydrogels as efficient solar generators for desalination. *Mater. Today Energy* **2020**, *16*, 100417.
3. Zhuang, P.; Li, D.; Xu, N.; Yu, X.; Zhou, L. Stable self-floating reduced graphene oxide hydrogel membrane for high rate of solar vapor evaporation under 1 sun. *Glob. Chall.* **2021**, *5*, 2000053.
4. Zhu, F.; Wang, L.; Demir, B.; An, M.; Wu, Z.; Yin, J.; Xiao, R.; Zheng, Q.; Qian, J. Accelerating solar desalination in brine through ion activated hierarchically porous polyion complex hydrogels. *Mater. Horiz.* **2020**, *7*, 3187–3195.
5. Wang, Z.; Yu, K.; Gong, S.; Mao, H.; Huang, R.; Zhu, Z. Cu_3BiS_3/MXenes with excellent solar-thermal conversion for continuous and efficient seawater desalination. *ACS Appl. Mater. Interfaces* **2021**, *13*, 16246–16258.
6. Guo, Y.; Lu, H.; Zhao, F.; Zhou, X.; Shi, W.; Yu, G. Biomass-derived hybrid hydrogel evaporators for cost-effective solar water purification. *Adv. Mater.* **2020**, *32*, 1907061.
7. Yin, J.; Xu, J.; Xu, W.; Liu, S.; Li, W.; Fang, Z.; Lu, C.; Xu, Z. Tuning the wettability of solar absorbers towards high-efficiency solar vapor generation. *Appl. Therm. Eng.* **2021**, *183*, 116224.
